# Rebound pain after regional anesthesia in orthopedic surgery: current evidence and clinical implications – a narrative review

**DOI:** 10.3389/fmed.2026.1930868

**Published:** 2026-10-16

**Authors:** Alexandra Elena Lazar, Oana Frandeș, Mircea Constantin Gherghinescu, Leonard Azamfirei

**Affiliations:** 1 Department of Anesthesia and Intensive Care, George Emil Palade University of Medicine, Pharmacy, Science and Technology, Targu Mures, Romania; 2 Emergency Clinical County Hospital, Targu Mures, Romania; 3 Doctoral School of Medicine and Pharmacy, George Emil Palade University of Medicine, Pharmacy, Science, and Technology, Targu Mures, Romania; 4 Department of Surgery, George Emil Palade University of Medicine, Pharmacy, Science and Technology, Targu Mures, Romania

**Keywords:** mechanism, orthopedic surgery, pain, rebound, regional anesthesia

## Abstract

Regional anesthesia is widely used in orthopedic surgery because it provides effective postoperative analgesia and reduces opioid requirements. However, increasing attention has been directed toward rebound pain, a phenomenon characterized by the abrupt emergence of severe pain following the resolution of a peripheral nerve block. Although frequently reported, rebound pain remains inconsistently defined, and its true incidence and clinical significance are still debated. Current evidence suggests that rebound pain is a multifactorial phenomenon influenced not only by block resolution itself but also by the transition from regional analgesia to systemic pain management. Variability in postoperative analgesic strategies, timing of rescue analgesia, patient expectations, and surgical factors may substantially affect both its occurrence and perceived severity. Differences in definitions and outcome measures across studies further contribute to the wide variation in reported incidence rates. Available data support several potential mechanisms, including the unmasking of ongoing postoperative nociceptive input after block resolution, inflammatory responses related to surgical tissue injury, and, in some cases, central sensitization. Clinical observations also indicate that inadequate multimodal analgesia and failure to anticipate the analgesic transition period may play a major role in the development of severe rebound pain episodes. Current evidence suggests that proactive analgesic planning, scheduled multimodal analgesia initiated before block resolution, intravenous dexamethasone, and prolonged regional anesthesia techniques may reduce the incidence or severity of rebound pain. However, the reported benefits are not consistent across all studies, and some interventions improved early analgesia or prolonged block duration without reducing rebound pain or opioid consumption. Interpretation remains limited by substantial clinical and methodological heterogeneity. Rebound pain appears to represent a complex interaction between physiological nociceptive processes and modifiable perioperative management factors rather than a simple consequence of peripheral nerve block resolution alone. Greater standardization of definitions and outcome measures is needed to better characterize its incidence, mechanisms, and clinical relevance in orthopedic surgery.

## 1 Introduction

Regional anesthesia has become an integral component of contemporary perioperative care in orthopedic surgery because of its ability to provide effective postoperative analgesia while reducing opioid requirements. Regional anesthetic techniques are incorporated into perioperative pathways for hip and knee arthroplasty (1). Parallel consensus work has sought to standardize nomenclature and anatomical descriptions for several regional-anesthesia blocks (2).

Despite these well-established analgesic benefits, growing attention has been directed toward rebound pain, characterized by a marked increase in pain after resolution of a peripheral nerve block. The phenomenon was quantified in the early clinical literature using the change in pain score associated with block resolution (3). Subsequent reviews have highlighted its potential association with increased analgesic requirements, sleep disturbance, reduced patient satisfaction, and unplanned healthcare utilization, particularly when block resolution occurs after discharge (4, 5).

Despite increasing clinical recognition, substantial uncertainty remains regarding the nature of rebound pain. The literature is characterized by considerable heterogeneity in definitions, reported incidence rates, assessment methods, and proposed pathophysiological mechanisms. It remains unclear whether rebound pain represents a distinct biological phenomenon, an unmasking of expected postoperative nociception, or, at least in part, a methodological artifact influenced by analgesic protocols and outcome assessment strategies.

Previous narrative reviews have described the definition, incidence, proposed mechanisms, clinical consequences, and prevention of rebound pain after peripheral nerve blockade, including its occurrence in orthopedic surgery (4–9). However, substantial heterogeneity remains in how recent randomized trials define rebound pain and evaluate preventive interventions. The present review therefore adds a structured reappraisal of contemporary randomized evidence, interpreted according to each study's original definition and outcome measures. Particular emphasis is placed on the transitional analgesic gap as a potentially modifiable contributor and on whether apparently conflicting intervention effects can be explained by differences in block duration, background multimodal analgesia, and the timing of transition from regional to systemic pain control.

This narrative review examines the definitions, incidence, proposed mechanisms, clinical predictors, and preventive strategies associated with rebound pain after regional anesthesia in orthopedic surgery. Recent randomized evidence is critically interpreted through the framework of the transitional analgesic gap, while distinguishing physiological mechanisms from potentially modifiable perioperative management factors.

## 2 Definition of rebound pain: current heterogeneity in the literature

One of the major challenges in interpreting the literature on rebound pain is the absence of a universally accepted definition. Although the phenomenon is generally described as a transient increase in pain following the resolution of a peripheral nerve block, substantial heterogeneity exists regarding the diagnostic criteria used across clinical studies. Consequently, reported incidence rates and the perceived effectiveness of preventive strategies are strongly influenced by the definition adopted, limiting direct comparison between studies and complicating evidence synthesis.

The most frequently used definition conceptualizes rebound pain as a clinically relevant increase in pain intensity after block resolution rather than as an absolute postoperative pain score. This operational definition was used as an interpretative framework for the present review and was not retrospectively imposed on studies that applied different definitions or outcome measures. Each study was interpreted according to its original definition of rebound pain. When rebound pain was not explicitly defined, outcomes such as opioid consumption or postoperative pain trajectory were considered indirect indicators rather than diagnostic criteria.

This approach reflects the characteristic transition from complete sensory blockade to restored nociceptive transmission and has become the predominant operational definition in recent orthopedic randomized trials (10–12). For the purpose of this review, rebound pain is defined as a clinically relevant increase in pain intensity following resolution of a previously effective peripheral nerve block. This operational definition was selected because it captures the dynamic nature of the phenomenon and is applicable across different surgical procedures, pain assessment scales, and analgesic protocols. By emphasizing the change in pain intensity rather than a predefined pain threshold, this definition characterizes rebound pain as a dynamic post-block pain phenomenon rather than simply an episode of severe postoperative pain.

Throughout this review, rebound pain refers exclusively to the clinically relevant increase in pain intensity following resolution of a previously effective peripheral nerve block. The transitional analgesic gap refers specifically to the interval between loss of regional analgesia and establishment of effective systemic analgesia and is considered a potential contributor to, rather than a synonym for, rebound pain. The term rebound analgesia is retained only as a search synonym in the literature search strategy and is not used as a separate clinical concept.

An alternative approach defines rebound pain according to the occurrence of severe postoperative pain after block resolution, most commonly using a Numeric Rating Scale (NRS) or Visual Analogue Scale (VAS) score ≥7. Although this methodology is simple and easily reproducible, it does not account for interindividual differences in pain perception and may fail to identify patients who experience a marked increase in pain that remains below the selected cutoff value (12, 13).

Attempts to standardize rebound pain assessment have been limited. Korkusuz et al. (2023) introduced the Modified Rebound Pain Score (MRPS), a composite outcome intended to evaluate rebound pain beyond a single pain intensity measurement. Nevertheless, this score has not been externally validated and has not been adopted by subsequent clinical studies, precluding its use as a standardized assessment tool. At present, the MRPS should therefore be regarded as an exploratory methodological proposal rather than an established definition of rebound pain (14).

Overall, the available evidence demonstrates that rebound pain is currently defined using heterogeneous methodological approaches rather than uniform diagnostic criteria. The operational definition adopted in this review—a clinically relevant increase in pain intensity following resolution of a previously effective peripheral nerve block—provides a consistent framework for interpreting the available evidence while acknowledging the methodological heterogeneity of the literature. Nevertheless, the development of an internationally accepted definition incorporating both the temporal relationship with block resolution and clinically meaningful pain outcomes should remain a priority for future research.

## 3 Methodological implications for measurement and comparison

An additional methodological challenge is the inherently subjective and individually variable nature of pain. Unlike physiological variables, pain is a multidimensional experience shaped by biological, psychological, social, and contextual factors (15–17).

Numerical pain scales may not fully reflect the patient's experience because pain intensity is influenced by anxiety, expectations, coping, previous pain, and individual pain modulation. The included trials consequently measured related but non-equivalent outcomes: change in pain intensity after block resolution (11, 12), pain at prespecified time points (10), severe pain above a predefined threshold (13), the composite Modified Rebound Pain Score (14), and indirect outcomes such as opioid consumption or rescue-analgesia timing (10–14).

Change scores may identify a marked escalation that remains below a severe-pain threshold, whereas an absolute NRS or VAS threshold cannot distinguish an abrupt post-block increase from continuously severe postoperative pain. Composite scores remain difficult to compare without external validation, and opioid consumption is a surrogate influenced by prescribing and rescue-analgesia protocols. These outcome differences must therefore be considered when interpreting incidence and intervention effects.

From a methodological perspective, rebound pain should therefore be interpreted as a patient-reported outcome rather than an objective physiological event. Future studies would benefit from incorporating multidimensional assessment tools that combine pain intensity with analgesic consumption, functional recovery, sleep quality, patient satisfaction, and the temporal relationship with block resolution. Such an approach would more accurately reflect the clinical impact of rebound pain and improve comparability between studies while acknowledging the intrinsically subjective nature of postoperative pain

## 4 Incidence according to surgery type and block technique

The reported incidence of rebound pain varies substantially according to the surgical procedure, regional anesthesia technique, and definition used.

The highest and most consistent incidence has been reported after shoulder surgery performed under single-shot interscalene block. Across randomized controlled trials, approximately one-third to more than 80% of patients in the control groups experienced rebound pain, whereas preventive strategies such as intravenous or perineural dexamethasone and multimodal periarticular injections reduced its incidence to approximately 20%–40% in several trials (12, 13, 18–20).

In upper-limb surgery, including distal radius, wrist, and hand procedures performed under supraclavicular or infraclavicular brachial plexus block, reported incidence was also highly variable. The highest rates were observed in patients receiving standard single-shot blocks without preventive strategies, while intravenous dexamethasone, continuous infraclavicular blockade, and selected local anesthetic combinations substantially reduced rebound pain (10, 11, 21, 22).

Evidence from knee surgery is less consistent. In unicompartmental knee arthroplasty, prolongation of block duration alone did not prevent rebound pain and was even associated with higher rebound pain scores in one study, whereas adding perineural methylprednisolone to an already optimized multimodal regimen did not provide additional benefit after total knee arthroplasty (23, 24).

For hip and foot/ankle surgery, fewer studies are available. Liposomal bupivacaine PENG block reduced rebound pain after hip fracture surgery, whereas observational data from ankle fracture surgery confirmed rebound pain as a clinically relevant problem after block resolution, although precise incidence estimates remain uncertain (25, 26).

Reported incidence is also strongly influenced by the definition of rebound pain. Studies defining rebound pain as any clinically relevant increase in pain after block resolution generally report higher incidence than those using threshold-based criteria (NRS/VAS ≥7), modified rebound pain scores, or composite outcomes combining pain intensity with opioid consumption (7, 14, 27). Consequently, differences in reported incidence should be interpreted in the context of both the surgical procedure and the methodology used to define rebound pain.

Reported incidence estimates vary substantially across studies, partly because of differences in surgical procedures, regional anesthesia techniques, postoperative analgesic protocols, and operational definitions of rebound pain. Table 1 summarizes the incidence reported by the included orthopedic studies according to these characteristics.

**Table 1:** Reported rebound pain among the included studies. NRS, Numeric Rating Scale; NPRS, Numeric Pain Rating Scale; VAS, Visual Analog Scale. Only studies reporting a numerical incidence of rebound pain were included.

Study	Surgical procedure	Regional anesthesia technique	Definition of rebound pain used in the study	Timing of pain assessment	Reported incidence
Kang et al. (2025) (11)	Wrist and hand surgery	Single-shot supraclavicular brachial plexus block	Severe pain after block resolution, defined as NRS ≥7	Before and after block resolution; follow-up to 24 h	Intravenous dexamethasone: 32%; control: 79%
Yao et al. (2025) (12)	Arthroscopic shoulder surgery	Single-shot interscalene block	Severe rebound pain, defined as NRS ≥7 after block resolution	After block resolution; follow-up to 24 h	Dexamethasone: 22%; no dexamethasone: 36%. Esketamine: 27%; no esketamine: 32%
Nobre et al. (2025) (13)	Shoulder surgery	Interscalene brachial plexus block	Sudden onset of moderate-to-severe pain, defined as VAS ≥7, without improvement after oral medication	During the post-block period; exact follow-up not reported	Control: 41.2%; dexamethasone: 28.6%; clonidine: 23.3%
Woo et al. (2021) (18)	Arthroscopic shoulder surgery	Single-shot interscalene block with ropivacaine	Severe pain after resolution of the interscalene block, defined using an NRS-based threshold	Before and after block resolution; follow-up to 48 h	Perineural dexamethasone: 37.1%; control: 82.9%
Et et al. (2023) (19)	Shoulder surgery	Single-shot interscalene brachial plexus block	Marked postoperative pain occurring after resolution of the block, assessed using the NPRS	After block resolution; follow-up to 48 h	Intravenous dexamethasone: 30.0%; control: 73.3%
Park et al. (2025) (20)	Arthroscopic rotator cuff repair	Single-shot interscalene brachial plexus block	Rapid increase in postoperative pain following resolution of the interscalene block, according to the study-specific pain assessment	12–48 h postoperatively	Multimodal shoulder injection: 18.8%; control: 65.7%
Jongkongkawutthi et al. (2025) (22)	Open reduction and internal fixation of distal radius fracture	Ultrasound-guided axillary brachial plexus block	Increase of more than 2 points from the patient's average postoperative pain score	Postoperative assessment; exact timing not reported	Bupivacaine–lidocaine–dexamethasone mixture: 8.6%; bupivacaine alone: 28.6%
Li et al. (2024) (23)	Unicompartmental knee arthroplasty	Combined femoral and sciatic nerve blocks	Study-specific composite rebound pain assessment following block resolution	12–36 h postoperatively	Perineural dexamethasone: 57.6%; ropivacaine alone: 32.4%
Wang et al. (2025) (26)	Hip fracture surgery	Pericapsular nerve group block	Transition from adequately controlled pain during the effective block period to severe pain, defined as NRS ≥7, following block resolution	0–72 h postoperatively	Liposomal bupivacaine: 10.0%; ropivacaine: 26.7%; saline: 43.3%
Lee et al. (2023) (28)	Arthroscopic rotator cuff repair	Single-shot interscalene block	Severe surgical-site pain after block resolution, defined as NRS ≥7	After block resolution; follow-up to 7 days	Intravenous dexamethasone: 20.0%; perineural dexamethasone: 44.4%

As shown in Table 1, the wide range of reported incidence values reflects not only differences in surgical and anesthetic practice but also considerable variability in the operational definition of rebound pain. Some studies used a threshold for severe pain, commonly NRS or VAS ≥7, whereas others used changes from baseline pain scores or study-specific composite assessments. Therefore, direct comparisons between incidence estimates should be interpreted cautiously

## 5 Pathophysiology: plausible mechanisms of rebound pain

### 5.1 Nociceptive input after block resolution

The restoration of nociceptive transmission following peripheral nerve block resolution is currently the best-supported clinical explanation for rebound pain, although a transient hyperalgesic contribution cannot be excluded. Peripheral nerve blocks suppress afferent transmission but do not prevent surgical injury, inflammation, or peripheral sensitization; nociceptive input therefore continues to develop during effective sensory blockade (5, 7).

As neural conduction recovers, this previously masked nociceptive input reaches the central nervous system, producing the rapid pain transition described as the “unmasking phenomenon” (4, 5, 7). Clinical observations and trials showing attenuation with prolonged regional analgesia or improved transitional pain management are compatible with this model, but direct mechanistic evidence remains limited (10, 12).

### 5.2 Central sensitization

Central sensitization has been proposed as a potential contributor to rebound pain, but current evidence does not support it as the primary mechanism. The abrupt onset of pain after peripheral nerve block resolution and its typically transient course are more consistent with restoration of postoperative nociceptive input and unmasking of ongoing surgical pain than with a sustained centrally mediated hyperalgesic state (5, 7).

Central sensitization refers to an activity-dependent increase in the excitability of neurons within central nociceptive pathways, leading to enhanced pain processing and reduced pain thresholds (29, 30). Surgical tissue injury generates persistent nociceptive input that may promote these processes, while regional anesthesia may attenuate—but not completely prevent—central sensitization by interrupting afferent transmission during surgery and the early postoperative period, providing one of the theoretical rationales for preventive analgesia (31, 32). However, direct mechanistic evidence linking rebound pain to central sensitization remains limited.

Thus, central sensitization should be viewed as a potential amplifying factor rather than the primary driver of rebound pain. The available clinical evidence is more consistent with restoration of postoperative nociceptive input following peripheral nerve block resolution as the principal explanation for rebound pain.

### 5.3 Inflammatory response

Inflammation is a biologically plausible contributor to rebound pain, but it should be interpreted as an amplifying mechanism rather than as an isolated cause. Surgical tissue injury initiates a local inflammatory response characterized by the release of prostaglandins, bradykinin, histamine, cytokines, and other mediators that sensitize peripheral nociceptors and lower their activation threshold. This peripheral sensitization increases the intensity of nociceptive signaling generated at the surgical site and contributes to the evolution of acute postoperative pain (33–35).

Peripheral nerve blockade interrupts afferent sensory transmission but does not abolish the inflammatory response induced by surgical trauma. Therefore, during the period of sensory blockade, inflammatory and nociceptive processes continue to evolve despite the absence of perceived pain. When local anesthetic concentrations decline and neural conduction recovers, sensory pathways are re-exposed to an inflamed tissue environment with heightened nociceptor excitability. This interaction provides a plausible explanation for the abrupt and intense pain that may occur after block resolution, particularly when the duration of the block is shorter than the trajectory of postoperative inflammatory pain.

The potential role of inflammation is indirectly supported by studies evaluating dexamethasone for prevention of rebound pain. Intravenous and perineural dexamethasone have reduced rebound pain in several trials, although findings are not uniformly positive (18, 36, 37). The observed effect may reflect systemic anti-inflammatory activity, prolongation of block duration, improved overlap between regional analgesia and the postoperative pain trajectory, or a combination of these mechanisms. Meta-analytic evidence supports an overall reduction in rebound-pain incidence after single-injection peripheral nerve blocks, but does not establish the relative contribution of anti-inflammatory effects and block prolongation (37).

Inflammation should be considered an important component of the multifactorial model of rebound pain. Ongoing postoperative inflammation amplifies peripheral nociceptive signaling, while recovery of sensory transmission permits this signal to reach the central nervous system. However, current evidence supports inflammation as a contributor to pain amplification rather than as a standalone mechanism explaining rebound pain.

### 5.4 Local anesthetic-associated hyperalgesia and neurotoxicity

Preclinical evidence provides direct support for the biological plausibility of a transient hyperalgesic component. Kolarczyk and Williams demonstrated that 0.5% ropivacaine sciatic nerve block in rats produced transient heat hyperalgesia as mechanical analgesia resolved, without a corresponding mechanical hyperalgesic response; persistent hyperalgesia or tactile allodynia was not demonstrated at two weeks (38). This modality-specific finding cannot establish that clinical rebound pain is caused by local anesthetic-induced hyperalgesia, but it should not be dismissed. Local anesthetic neurotoxicity is also dependent on concentration, dose, and exposure, and experimental injury may involve mitochondrial dysfunction, calcium dysregulation, apoptosis, and inflammatory pathways (39). Thus, the animal findings support a potential mechanistic contribution, whereas current clinical evidence remains insufficient to determine its magnitude or to regard it as the dominant explanation for rebound pain. Figure 1 summarizes the above mentioned pathophysiological mechanisms.

**Figure 1:**
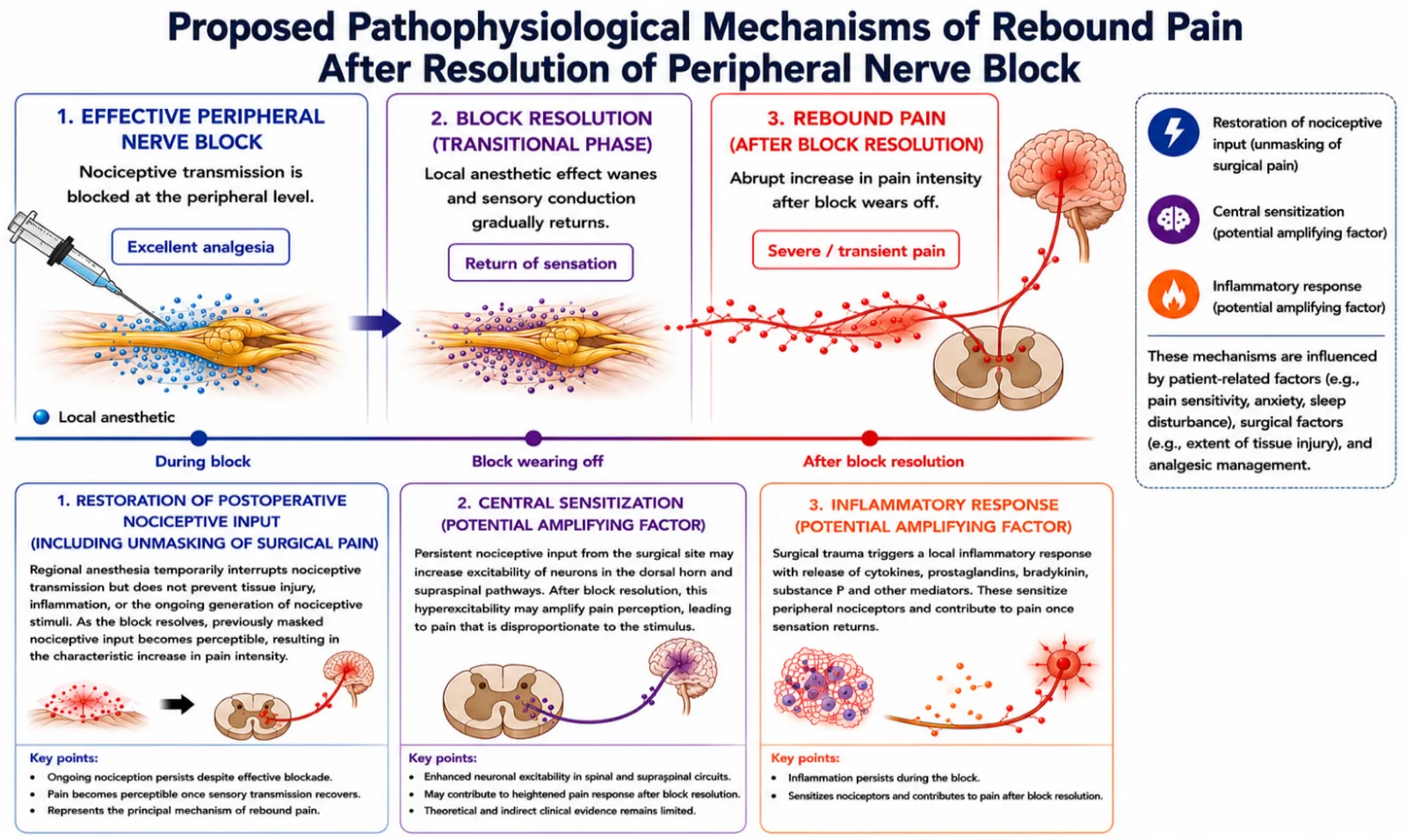
Potential pathophysiological mechanism of rebound pain – a proposed conceptual model.

## 6 Methodological considerations: the transitional analgesic gap as a contributor to rebound pain

The transitional analgesic gap is the interval between loss of regional analgesia and establishment of effective systemic pain control. Its severity may be influenced by block duration, the postoperative pain trajectory, delayed systemic analgesia, and patient expectations, particularly when block resolution occurs after discharge (5, 9).

The transitional analgesic gap should therefore be interpreted as one potentially modifiable contributor to rebound pain rather than as synonymous with it. Rebound pain itself remains the clinically relevant increase in pain following block resolution and may also be influenced by surgical nociceptive burden, inflammatory processes, and patient-related factors. This distinction helps explain the heterogeneity of reported outcomes, while the corresponding clinical interventions are discussed separately in the Prevention Strategies section.

## 7 Clinical predictors for rebound pain

Potential predictors of rebound pain should be interpreted according to the type of evidence supporting them. Randomized trials primarily provide evidence regarding modifiable anesthetic and analgesic interventions, whereas most patient- and surgery-related predictors have been identified through observational studies or secondary analyses. The principal multivariable evidence derives from the cohort reported by Barry et al. (27). Adjusted effect estimates and confidence intervals are provided in Supplementary Table S2 and represent associations rather than causal effects.

### 7.1 Surgical factors

Evidence concerning surgical predictors is derived predominantly from observational data and comparisons across heterogeneous studies. Among the adjusted predictors, surgery involving bone rather than soft tissue was associated with rebound pain (27). Shoulder and other major orthopedic procedures have also frequently been associated with a high incidence of rebound pain, although these comparisons may be confounded by differences in block technique, postoperative analgesic protocols, and outcome definitions (4, 5). Surgical procedure should therefore be considered a risk marker rather than an independently established causal factor.

### 7.2 Anesthetic and analgesic factors

Among anesthetic and management-related variables, absence of perioperative intravenous dexamethasone was independently associated with rebound pain (27). Other preventive interventions have produced inconsistent procedure-specific effects and are discussed in Section 9; they should not be interpreted as universal clinical predictors.

### 7.3 Patient-related factors

Younger age and female sex were independently associated with rebound pain (27). Diabetes, pain catastrophizing, and preexisting pain were not evaluated as independent predictors in that cohort. In the randomized trial by Touil et al., however, severe preoperative pain and high pain catastrophizing were identified as secondary predictive factors; these exploratory associations require confirmation in larger prospective cohorts (40).

## 8 Literature search strategy

A structured literature search was performed in PubMed on 12 June 2026 to identify studies evaluating rebound pain following regional anesthesia. PubMed was selected because of its comprehensive coverage of peer-reviewed biomedical and clinical literature and was considered appropriate for the focused scope and narrative design of this review. Title and abstract screening and eligibility assessment were performed by one author. The search strategy combined the terms (“rebound pain” OR “rebound analgesia” OR “pain after nerve block” OR “post-block pain”) AND [“Anesthesia, Conduction”[MeSH] OR “Peripheral Nerve Block”[MeSH] OR “regional anesthesia”] AND (orthopedic OR surgery). The search was restricted to articles published between June 2021 and June 2026. This five-year period was chosen to capture contemporary evidence reflecting current regional anesthesia techniques, postoperative analgesic protocols, definitions of rebound pain, and emerging preventive strategies. In the search strategy, the term ‘rebound analgesia’ was used solely as a retrieval synonym for rebound pain and is not treated as a distinct clinical entity in this review.

Studies were eligible for inclusion if they were published in English and directly addressed the definition, incidence, pathophysiology, risk factors, prevention, or clinical implications of rebound pain following regional anesthesia. The primary evidence base comprised studies conducted in orthopedic surgical populations. However, selected non-orthopedic studies were also included when they evaluated mechanisms or pharmacological interventions potentially transferable to orthopedic regional anesthesia, particularly strategies involving local anesthetic adjuvants or systemic agents. These studies were used as supportive evidence and not to estimate the incidence of rebound pain in orthopedic surgery or to draw procedure-specific orthopedic conclusions. Randomized controlled trials, prospective and retrospective observational studies, systematic reviews, and narrative reviews were considered. Studies were excluded if they were not published in English, did not directly address rebound pain following regional anesthesia, or did not provide evidence relevant to the objectives of the review.

The search identified 29 records. After title and abstract screening, two articles were excluded: one because it was published in German and one because it did not directly address rebound pain following regional anesthesia. Consequently, 27 studies were included in the present narrative review. Given the narrative design of the review, the diagram is not intended to represent a PRISMA-compliant systematic review process. Generative AI was used only for language and editorial support and was not treated as a source of evidence. Every reference, bibliographic detail, and citation-to-claim alignment was independently verified by the authors against the corresponding primary publication.

Given the narrative nature of this review, the included studies were analyzed qualitatively, with particular emphasis on methodological approaches, definitions of rebound pain, proposed mechanisms, risk factors, preventive strategies, and clinical implications. A quantitative synthesis was not considered appropriate because of substantial clinical and methodological heterogeneity among the included studies, particularly regarding surgical procedures, regional anesthesia techniques, postoperative analgesic protocols, outcome measures, and definitions of rebound pain. Therefore, the findings should be interpreted as a qualitative and inherently interpretative synthesis rather than as pooled estimates of incidence or treatment effect.

The 27 included studies constituted the contemporary evidence base identified through the structured PubMed search. Additional publications were identified separately to provide historical, conceptual, methodological, or mechanistic background. Sort et al. (25), published in 2019, was retained only as historical observational context and was not counted among the 27 included studies or included in the evidence map. During revision, targeted reference checking identified the directly relevant randomized trials by Touil et al. (40) and Fang et al. (41), which were incorporated into the qualitative intervention synthesis and clearly distinguished from the original structured-search yield. Fang et al. was published in February 2021, before the lower boundary of the stated search window. The historical preclinical study by Kolarczyk and Williams was used only to inform the mechanistic discussion. The complete evidence map and quantitative study characteristics for the 27 studies identified by the structured search are presented in Supplementary Tables S1, S2 (10–14, 18–24, 26, 28, 42–54).

## 9 Current clinical evidence on rebound pain: analysis of randomized controlled trials

Because the included trials used different definitions and outcome measures, comparisons of rebound-pain incidence and preventive efficacy were made within the context of each study’s original operationalization rather than by applying the definition adopted in this review retrospectively.

Recent randomized controlled trials have shifted the focus from describing rebound pain to evaluating preventive strategies, although substantial heterogeneity in surgical procedures, regional techniques, analgesic protocols, and outcome definitions limits direct comparison between studies. The evidence summarized in Supplementary Table S1 is primarily derived from orthopedic surgery. Selected non-orthopedic trials were retained when they provided relevant pharmacological or mechanistic evidence potentially applicable to rebound pain in orthopedic regional anesthesia. Their surgical setting is explicitly identified in the table, and their findings are interpreted as supportive rather than procedure-specific evidence.

### 9.1 Intravenous or perineural dexamethasone

Dexamethasone is the most extensively investigated pharmacological strategy for preventing rebound pain, but the findings are not uniformly positive. Several randomized trials reported that intravenous dexamethasone reduced rebound pain severity, opioid consumption, or sleep disturbance, while perineural administration also showed benefit in some surgical populations (11, 12, 18, 21, 28). However, Nobre et al. found no statistically significant reduction in rebound pain with dexamethasone or clonidine after interscalene block for shoulder surgery (13). Moreover, Li et al. reported that perineural dexamethasone prolonged block duration and improved early analgesia after unicompartmental knee arthroplasty but was associated with a higher incidence of rebound pain (23). Other studies demonstrated only modest improvements in pain-related outcomes without corresponding reductions in opioid consumption. These findings indicate that prolongation of sensory blockade does not necessarily prevent rebound pain and that the effect of dexamethasone may depend on the route and dose of administration, surgical procedure, block technique, background multimodal analgesia, and definition and timing of rebound pain assessment.

### 9.2 Continuous regional techniques and prolonged analgesia

Strategies extending analgesic coverage beyond the immediate postoperative period have generally been associated with attenuation of rebound pain, although the magnitude and consistency of this benefit vary across studies. Continuous infraclavicular blockade reduced early rebound pain after distal radius fixation, while liposomal bupivacaine lowered rebound pain and opioid requirements after hip surgery (10, 26).

### 9.3 Dexmedetomidine

Perineural dexmedetomidine improved early postoperative analgesia and reduced rebound pain after shoulder arthroscopy, although evidence is currently limited to a small randomized trial (42).

### 9.4 Opioids

Opioid-based preventive strategies have not shown similar efficacy. Pre-emptive oral hydromorphone failed to reduce rebound pain severity, pain trajectory, or opioid consumption after shoulder arthroscopy (43).

### 9.5 Ketamine and esketamine

The most directly relevant ketamine trial was conducted by Touil et al. in 109 ambulatory patients undergoing upper-limb surgery with axillary plexus block. A single intraoperative anti-hyperalgesic dose of intravenous ketamine (0.3 mg kg−1) did not reduce the incidence or intensity of severe rebound pain; temporal summation and Central Sensitization Inventory scores also did not differ between patients with and without rebound pain (40). Intravenous esketamine likewise did not reduce rebound pain after interscalene block for shoulder surgery, although benefit was reported after thoracic paravertebral block (12, 44). These findings do not support a consistent preventive effect of ketamine-based strategies and suggest that any benefit may depend on the surgical and regional-anesthesia setting.

### 9.6 Other interventions

Surgeon-administered multimodal periarticular injections reduced rebound pain and opioid requirements after rotator cuff repair (20). Perineural methylprednisolone and clonidine did not provide a clear additional benefit in individual trials (13, 24), whereas modifications of local-anesthetic composition produced procedure-specific findings (22, 46). In a distal radius fracture trial, adding perineural dexamethasone modestly prolonged block duration but did not produce a clinically important overall improvement in postoperative pain (45).

Overall, available evidence suggests that rebound pain may be modifiable through strategies aimed at maintaining continuity of analgesia. Intravenous dexamethasone and prolonged regional techniques may reduce rebound pain in some clinical settings; however, their benefits are not consistent across all studies, procedures, or outcome measures. Interpretation remains limited by heterogeneous definitions, analgesic protocols, and assessment method.

## 10 Prevention strategies

### 10.1 Scheduled multimodal analgesia

Scheduled multimodal analgesia represents a central component of rebound pain prevention. Rather than waiting for pain to return, acetaminophen, NSAIDs or COX-2 inhibitors, and rescue opioids when indicated should be initiated while the peripheral nerve block is still providing effective analgesia. This proactive approach aims to minimize the transitional analgesic gap between block resolution and systemic analgesic efficacy (5, 8).

### 10.2 Prolongation of regional analgesia

Maintaining analgesic coverage during the period of greatest surgical pain is equally important. Continuous peripheral nerve catheters have been associated with improved postoperative pain control compared with opioid-based analgesia (55). In a randomized trial after distal radius fixation, continuous infraclavicular blockade specifically reduced early rebound pain (10).

### 10.3 Rational use of adjuvants

Pharmacological adjuvants may reduce rebound pain by prolonging block duration and modulating perioperative inflammation. Systematic review evidence and several randomized trials suggest that intravenous or perineural dexamethasone may reduce rebound pain after peripheral nerve blockade (18, 28, 37, 41). Nevertheless, individual trials have reported no significant benefit or an increased incidence of rebound pain despite prolonged block duration (13, 23). Therefore, dexamethasone should be regarded as a potentially beneficial component of a broader multimodal analgesic strategy rather than as a uniformly effective preventive intervention.

Together, these strategies aim to maintain continuity of analgesia throughout the transition from regional to systemic pain control. A planned multimodal approach combining scheduled analgesia, prolonged regional techniques when appropriate, and rational use of adjuvants appears to offer the most clinically coherent strategy for minimizing the impact of rebound pain.

## 11 Practical considerations according to the care setting

Prevention strategies should be adapted to the perioperative setting. In hospitalized patients, the transition from regional to systemic analgesia can be supervised through scheduled multimodal analgesia, timely administration of rescue medication, and regular pain assessment during block resolution. In contrast, ambulatory patients require particular attention, as peripheral nerve block resolution frequently occurs after discharge, when healthcare professionals are no longer available to guide analgesic management. Patients should therefore receive clear verbal and written instructions regarding the expected duration of the block, the anticipated return of sensation, the importance of initiating scheduled analgesics before block resolution rather than waiting for severe pain to develop, and the circumstances requiring medical advice. These recommendations are supported primarily by contemporary narrative reviews and expert guidance rather than by dedicated trials of patient education (4, 8).

## 12 Methodological limitations of the current evidence

The current evidence on rebound pain is limited by substantial methodological heterogeneity. The literature search was restricted to PubMed and was not extended to Embase, Cochrane CENTRAL, or Web of Science. Therefore, relevant studies indexed exclusively in other databases may have been missed. Reliance on a single database represents an important limitation of the present narrative review. In addition, title and abstract screening and eligibility assessment were conducted by a single author, which may have increased the risk of selection error and subjective judgment. The absence of a standardized definition and the use of heterogeneous outcome measures make comparisons across studies difficult and preclude reliable estimates of its incidence (10, 11, 14, 18). Interpretation of the available data is further complicated by considerable variation in perioperative analgesic protocols, including multimodal analgesic strategies, regional anesthesia techniques, and timing of analgesic administration (10, 18, 20, 23).

The diversity of surgical procedures, each with distinct postoperative pain trajectories and analgesic requirements, further limits the generalizability of the findings (14, 44). The inclusion of selected non-orthopedic studies represents an additional limitation. Although these studies provide potentially transferable pharmacological or mechanistic evidence, differences in surgical injury, baseline pain trajectories, patient populations, regional anesthesia techniques, and perioperative analgesic protocols limit the direct applicability of their findings to orthopedic surgery. These studies were considered supportive evidence and were not used to estimate orthopedic-specific incidence or to formulate procedure-specific conclusions.

In addition, pain is an inherently subjective experience, making its measurement and comparison challenging. Finally, dedicated mechanistic studies remain scarce, and current hypotheses are largely based on indirect clinical observations rather than direct experimental evidence (5, 7). Conflicting findings across preventive trials may also reflect clinically important differences in surgical procedures, baseline postoperative pain trajectories, regional anesthesia techniques, adjuvant routes and doses, and background multimodal analgesic protocols. In addition, differences in the definitions of rebound pain, assessment time points, and reported outcomes may lead the same intervention to appear effective in one study but ineffective in another. Consequently, reductions in pain intensity, prolongation of block duration, and reductions in opioid consumption should not be interpreted as interchangeable outcomes.

Future studies should adopt consensus definitions, standardized outcome measures, and procedure-specific designs to improve comparability and strengthen the evidence base.

## 13 Conclusion

Rebound pain should be understood as a clinically relevant increase in pain following block resolution rather than as a distinct complication caused by regional anesthesia itself. Its severity reflects the interaction among surgical nociceptive burden, inflammatory amplification, block characteristics, patient susceptibility, and continuity of analgesic care. Current evidence supports anticipating block resolution, initiating scheduled multimodal analgesia before pain escalates, considering prolonged regional techniques when appropriate, and providing patients with clear instructions for the transition period. However, no single preventive intervention is uniformly effective, and management should remain procedure-specific and individualized.

Future research should prioritize a consensus definition developed through formal methods, such as a Delphi process, and a core outcome set that combines pain intensity and trajectory with analgesic consumption, sleep disturbance, functional recovery, and timing relative to block resolution. Adequately powered, procedure-specific trials should use standardized background analgesia and prespecified assessment intervals. Mechanistic studies integrating temporal sensory testing with relevant clinical or biological measures are also needed to distinguish the unmasking of expected postoperative nociception from genuine local anesthetic-associated hyperalgesia. These steps would improve comparability across studies and permit more reliable estimates of incidence, predictors, and intervention effects.
