# Supplementary material for: Rebound pain after regional anesthesia in orthopedic surgery: current evidence and clinical implications – a narrative review
Source: Front Med (Lausanne). 2026 Oct 16;13:1930868. doi: 10.3389/fmed.2026.1930868 (PMC13720677; doi:10.3389/fmed.2026.1930868)
Supplement: Supplementary file 1 [file Datasheet1.pdf]

No	Author, year	Study design	Surgery / population	Orthopedic surgery	Regional anesthesia / block	Intervention / comparator	Rebound pain role	Main results from abstract	Conclusion for narrative review
1	Hussain, 2025 [21]	RCT	Upper extremity: distal radius ORIF and/or carpometacarpal arthroplasty	Yes	Supraclavicular brachial plexus block	IV dexamethasone vs IV+perineural dexamethasone vs control	Secondary outcome	IV dexamethasone and IV+perineural dexamethasone prolonged sensory block vs control (21.3 h and 20.6 h vs 16.8 h) and reduced 24-h pain, opioid consumption, and incidence of rebound pain vs control. Adding perineural dexamethasone to IV dexamethasone gave no additional benefit.	IV dexamethasone alone appears sufficient; combined systemic+perineural route did not show additive/synergistic benefit.
2	Uppal 2024 [41]	Two-centre double-blind placebo-controlled RCT	Arthroscopic shoulder surgery	Yes	Single-shot interscalene block + general anesthesia	Oral hydromorphone 2 mg at 6 h vs placebo	Primary outcome related to rebound severity	Hydromorphone did not reduce worst pain within 24 h vs placebo (6.5 vs 5.9; mean difference 0.6; 95% CI -0.5 to 1.8). No difference in pain trajectory, opioid use, nausea/vomiting.	Pre-emptive hydromorphone 6 h after ISB did not reduce rebound pain severity.
3	Yao 2025 [12]	Patient/outcome-assessor-blinded 2x2 factorial RCT	Arthroscopic shoulder surgery	Yes	Single-shot interscalene ropivacaine block	IV esketamine and/or IV dexamethasone vs placebo combinations	Co-primary outcome	Rebound pain occurred in 32%. Dexamethasone reduced pain score increase and severe rebound pain (22% with dexamethasone vs 36% without; RR 0.62; P=0.016). Esketamine alone did not reduce severe rebound pain.	IV dexamethasone, but not low-dose esketamine, reduced incidence and severity of rebound pain after ISB.
4	Kang 2025 [11]	Randomized placebo-controlled trial	Wrist and hand surgery	Yes	Supraclavicular brachial plexus block	Preoperative IV dexamethasone 0.11 mg/kg vs saline	Primary outcome	Pain score increase after block resolution was lower with dexamethasone (4.7 vs 7.3; P<0.001). Rebound pain incidence was lower (32% vs 79%; P<0.001) and 24-h opioid use was	IV dexamethasone significantly reduced rebound pain and opioid consumption after wrist/hand surgery under

								lower (25 vs 72 mg; P<0.001).	supraclavicular block.
5	Wang 2025 [26]	RCT	Hip fracture surgery in older patients	Yes	PENG block	Liposomal bupivacaine vs ropivacaine vs saline	Primary/explicit rebound pain outcome	Rebound pain incidence was lower in liposomal bupivacaine and ropivacaine groups than saline. NRS AUC over 72 h was lowest with liposomal bupivacaine (2.053 vs 3.600 vs 4.880; P<0.0001). No difference in adverse events.	Liposomal bupivacaine PENG block reduced rebound pain, PCA demand, and improved satisfaction while preserving quadriceps function.
6	Et 2023 [19]	Prospective randomized controlled trial	Shoulder surgery	Yes	Interscalene block + general anesthesia	Multimodal analgesia with IV dexamethasone vs no dexamethasone	Primary/explicit rebound pain outcome	Rebound pain incidence was lower with dexamethasone than control (abstract reports 30% vs 73.3%; P=.001). NPRS after ISB resolution was lower (5 vs 8; P<.001). Sleep disturbance and QoR-15 improved.	IV dexamethasone added to multimodal analgesia with ISB reduced rebound pain and improved sleep/quality of recovery.
7	Wu 2025 [48]	RCT	Nuss procedure in children	No	Ultrasound-guided paravertebral block	Liposomal bupivacaine vs non-liposomal bupivacaine	Secondary outcome	Liposomal bupivacaine reduced morphine equivalents by about 23% over 72 h and reduced rebound pain incidence (13.0% vs 29.1%; RR 0.46; P=0.039). Sleep quality was better; adverse events similar.	Non-orthopedic study; supports long-acting local anesthetic strategies for reducing rebound pain after paravertebral block.
8	Lee 2023 [39]	RCT	Arthroscopic rotator cuff tear repair	Yes	Interscalene brachial plexus block	Perineural vs IV dexamethasone 5 mg	Primary outcome	Perineural dexamethasone prolonged ISB duration (19.9 vs 15.1 h) but pain increase after block	IV dexamethasone was more favorable than perineural dexamethasone for

								resolution was greater than IV dexamethasone (4.9 vs 4.0; P=0.043). Rebound pain was higher with perineural route (44.4% vs 20.0%; P=0.028).	rebound pain and sleep disturbance despite shorter block duration.
9	Charier 2021 [49]	Randomized controlled trial	Total hip replacement	Yes	Spinal anesthesia + lumbar plexus block vs GA + PCA or GA + lumbar plexus block	Regional anesthesia/opioid-free analgesia vs GA/opioid strategies	Not rebound pain; sleep rebound phenomenon	GA groups had REM sleep reduction on postoperative night 1 followed by REM rebound on nights 2-3 and increased AHI/arousals. Spinal + lumbar plexus block preserved sleep architecture and breathing.	Not a rebound-pain study; relevant only indirectly to regional anesthesia and postoperative recovery.
10	Li 2024 [23]	Double-blind RCT	Unicompartmental knee arthroplasty	Yes	Sciatic + femoral nerve blocks	Ropivacaine + perineural dexamethasone vs ropivacaine alone	Primary outcome	Adjuvant group had prolonged block and lower early pain at 12-20 h, but higher rebound pain incidence and score; later pain scores at 32-36 h were higher. CRP was lower.	Perineural steroid/adjuvant prolonged block but worsened rebound pain; the abstract contains a wording inconsistency between dexamethasone in title and dexmedetomidine in discussion.
11	Lee 2023 [10]	Prospective RCT	Distal radius fracture fixation	Yes	Single-shot infraclavicular brachial plexus block; continuous infraclavicular block in one arm	BPB only vs BPB + IV PCA vs BPB + continuous block	Primary/explicit rebound pain outcome	At 9 h, pain was higher with BPB only than IV PCA and continuous block. At 12 h, pain was higher with BPB only than continuous block. Total opioid equivalent consumption was highest in IV PCA; continuous block did not reduce total opioid consumption vs BPB only.	Continuous infraclavicular block controlled early rebound pain after distal radius fixation without increasing opioid consumption, unlike IV PCA.

12	Park 2025 [20]	Prospective randomized controlled trial	Arthroscopic rotator cuff repair	Yes	Interscalene brachial plexus block	Surgeon-administered multimodal shoulder injections vs saline	Primary and secondary outcomes	VAS at 12 h favored injection group (2.7 vs 4.1; P<.001). Rebound pain incidence was lower (18.8% vs 65.7%; P<.001). Pain from 12-48 h and fentanyl use over 48 h were lower; no injection-related complications.	Multimodal shoulder injections reduced rebound pain and opioid use after rotator cuff repair under ISBPB.
13	Huan 2025 [40]	Prospective randomized double-blind trial	Shoulder arthroscopy	Yes	Interscalene block	Perineural dexmedetomidine + ropivacaine vs ropivacaine alone	Primary/explicit rebound pain outcome	Perineural dexmedetomidine reduced rebound pain incidence, lowered pain at 12 and 24 h, delayed first analgesic request, reduced PCA sufentanil use, and decreased first-night sleep disturbance and nausea/vomiting.	Perineural dexmedetomidine added to ISB appeared beneficial for reducing rebound pain and improving early multimodal analgesia.
14	Zeng 2024 [42]	Prospective randomized double-blind placebo-controlled trial	Thoracoscopic lobectomy	No	Thoracic paravertebral block	Intraoperative IV esketamine vs saline	Primary/explicit rebound pain outcome	Esketamine reduced NRS at 24 and 48 h, reduced rebound pain incidence over 7 days, and lowered sufentanil use over 48 h. Overall postoperative recovery did not differ.	Non-orthopedic study; IV esketamine reduced rebound pain after thoracic paravertebral block.
15	Korkusuz 2023 [14]	RCT	Inguinal hernia surgery	No	Ilioinguinal/iliohypogastric nerve block	IV dexamethasone added to multimodal analgesia vs saline	Primary/explicit rebound pain outcome	Modified rebound pain scores were lower with dexamethasone at rest and with motion (both P=0.001). First 24-h tramadol use was lower (45.17 vs 95 mg; P<0.001).	Non-orthopedic study; IV dexamethasone with IIN/IHN block reduced rebound pain scores and opioid consumption.

16	Akaravinek 2023 [24]	RCT	Fast-track total knee arthroplasty	Yes	Peripheral nerve blocks + periarticular injection + IV dexamethasone	Perineural methylprednisolone added to PNBs vs no methylprednisolone	Secondary outcome	No difference in moderate-to-severe pain during first 12 postdischarge days (70% vs 69.2%; P=0.941). No difference in NRS scores, rebound pain incidence, or function; later moderate-to-severe pain was higher in methylprednisolone group.	Adding perineural methylprednisolone to a multimodal regimen with IV dexamethasone did not improve rebound pain or prolong analgesia after fast-track TKA.
17	Kim 2023 [44]	Randomized double-blind study	Distal radius fracture volar plating	Yes	Supraclavicular brachial plexus block	Ropivacaine+dexamethasone vs ropivacaine alone	Pain pattern / rebound-related	Dexamethasone produced clinically meaningful pain reduction only at 8 h (1.9 vs 4.7; P<0.001), prolonged block by ~3 h, but did not reduce fentanyl use or antiemetic use. Pain peaked at 12 h in both groups.	Dexamethasone modestly prolonged block and improved early pain but did not meaningfully change overall postoperative pain or opioid use.
18	Sellbrant 2021 [50]	Prospective randomized study	Distal radius fracture repair	Yes	Supraclavicular block	Mepivacaine vs ropivacaine vs general anesthesia	Postoperative pain / rebound-risk context	Mepivacaine SCB had lower 24-h NRS and lower day 0-3 opioid use than ropivacaine SCB. Unplanned healthcare contacts were highest with ropivacaine (39.3%). Authors caution that longer block duration may be associated with worse pain progression after resolution.	Suggests long-acting local anesthetic benefit must be balanced against rebound pain after block resolution.
19	Kim 2022 [51]	RCT	Shoulder arthroplasty	Yes	Interscalene brachial plexus block	Intraoperative remifentanil vs control	Indirect / not primary rebound pain	Remifentanil increased postoperative analgesic consumption over 24 h; total morphine-equivalent analgesic use was higher and hypotension more	Remifentanil may worsen postoperative analgesic requirements after shoulder

								frequent. Average NRS, PONV, and delirium were similar.	arthroplasty with ISB; rebound pain was not the main endpoint.
20	Woo 2021 [18]	RCT	Arthroscopic shoulder surgery	Yes	Single-shot interscalene block with ropivacaine	Perineural dexamethasone 5 mg vs control	Primary outcome	Pain increase after ISB resolution was lower with dexamethasone (4.5 vs 6.9; P<0.001). Rebound pain incidence was lower (37.1% vs 82.9%; P<0.001). Sleep disturbance was lower.	Perineural dexamethasone smoothed block resolution and reduced rebound pain and sleep disturbance.
21	Lee 2021 [52]	RCT	Arthroscopic rotator cuff repair	Yes	Suprascapular + axillary nerve block	Dexmedetomidine added to SSNB/ANB vs block alone	Secondary/indirect rebound pain outcome	Dexmedetomidine group had lower VAS and higher satisfaction within 48 h. Timing of rebound pain was later (36 h vs 23 h; P=0.007). Six patients in each group developed rebound pain.	Dexmedetomidine may delay rebound pain timing and improve early analgesia after rotator cuff repair.
22	Nobre 2025 [13]	Randomized clinical trial	Shoulder surgery	Yes	Interscalene block	Perineural dexamethasone vs clonidine vs no additive	Primary outcome	Rebound pain incidence was not significantly different between groups (control 41.2%, dexamethasone 28.6%, clonidine 23.3%; P=0.22). No significant differences in time to first pain or severity.	Perineural dexamethasone or clonidine did not significantly prevent rebound pain in this trial, despite numerically lower incidence.
23	Kasper 2025 [53]	Prospective randomized controlled blinded study	Distal radius fracture fixation	Yes	Supraclavicular block with ropivacaine	Perineural/direct dexamethasone vs IV/peripheral dexamethasone	Duration and rebound pain	Direct perineural dexamethasone changed block fade, onset of recovery, and block resolution compared with IV dexamethasone. It did not prove lower	Route of dexamethasone affected block duration/rebound profile but not clearly pain scores

								postoperative pain scores or opioid use within 24 h.	or opioid consumption.
24	Keller 2025 [54]	Prospective double-blind randomized placebo-controlled trial	Open sublay mesh hernia repair	No	Oblique subcostal TAP block	Ropivacaine TAP block vs placebo	Not primary rebound pain; rebound explanation	Ropivacaine group used more morphine by evening of POD2 (39 vs 24 mg; P=0.04), despite slightly less PACU morphine; later maximum pain was higher. Authors suggest rebound pain might explain excess opioid use.	Non-orthopedic; TAP block did not provide sustained opioid reduction and may have contributed to rebound-type opioid use.
25	Jongkongkawutthi 2025 [22]	Prospective randomized trial	Distal radius surgery	Yes	Axillary brachial plexus block	Bupivacaine-lidocaine-dexamethasone mixture vs bupivacaine alone	Secondary outcome	Motor and sensory recovery times were not significantly improved by the mixture. Pain scores were similar, but rebound pain incidence was lower with mixture (8.6% vs 28.6%; P=0.031).	LA mixture with dexamethasone did not improve motor recovery but reduced rebound pain incidence.
26	Aguilera 2025 [45]	RCT	Upper limb surgery	Yes	Infraclavicular brachial plexus block	0.5% bupivacaine vs 0.25% bupivacaine + 1% lidocaine; both with epinephrine+dexamethasone	Secondary outcome	0.5% bupivacaine prolonged motor block, sensory block, and postoperative analgesia compared with bupivacaine-lidocaine mixture, but had slower onset. No other significant differences were detected.	This regional anesthesia RCT recorded rebound pain but abstract does not report a significant rebound-pain difference.
27	Hillesheim 2021 [55]	Prospective randomized controlled trial	Arthroscopic rotator cuff repair	Yes	Single-shot interscalene block vs periarticular liposomal bupivacaine injection	PAI liposomal bupivacaine vs ISB	Indirect / rebound discussed in conclusion	ISB reduced pain and opioid use on surgery day vs PAI, but opioid use was higher with ISB on POD1 and POD2. Cumulative opioid use and 12-week	ISB gives superior immediate analgesia but rebound pain after block resolution may drive higher opioid use on

								outcomes were similar. PAI cost was lower.	POD1-2; PAI may be an alternative.

Supplementary Material

Supplementary Table 2. Methodological characteristics and quantitative outcomes of the included clinical studies

Author, year	Sample size	Primary endpoint	Follow-up	Effect estimate / principal quantitative result	95% CI	Key methodological limitations
**Hussain, 2025 [21]**	NR	Duration of sensory block	24 h	Sensory block: IV 21.3 h; IV+PN 20.6 h; control 16.8 h; both dexamethasone groups also reduced 24-h pain, opioid use and rebound pain	NR	Single study; rebound pain was secondary; three-group comparisons and route-specific effects limit precision.
**Uppal, 2024 [41]**	NR	Worst pain within 24 h after block	24 h	MD 0.6 points (hydromorphone vs placebo)	-0.5 to 1.8	Two-centre trial but limited to one opioid dose and one surgical setting; follow-up for the primary outcome was 24 h.
**Yao, 2025 [12]**	160	Pain-score increase after block resolution and severe rebound pain	24 h	Dexamethasone: severe rebound pain 22% vs 36%; RR 0.62. Esketamine: 27% vs 32%; RR 0.84	Dexamethasone: NR; esketamine: 0.50 to 1.41	Factorial trial; low-dose esketamine; interaction and multiple co-primary comparisons complicate interpretation.
**Kang, 2025 [11]**	56	Change in pain score before vs after block resolution	24 h	Pain increase 4.7 vs 7.3; rebound pain 32% vs 79%; 24-h opioid use 25 vs 72 mg	NR	Small, single-centre trial; short follow-up; several secondary outcomes without reported CIs.
**Wang, 2025 [26]**	90	Postoperative pain trajectory/rebound pain	72 h	Rebound pain: 10% with liposomal bupivacaine vs 43% with saline; NRS AUC 2.053 vs 3.600 vs 4.880	NR	Single-centre, three-arm trial in older hip-fracture patients; procedure- and formulation-specific generalizability.
**Et, 2023 [19]**	60	Incidence and severity of rebound pain after ISB resolution	48 h; QoR to day 7	Rebound pain 30.0% vs 73.3%; median post-resolution NPRS 5 vs 8	NR	Single-centre study; small sample; no reported CI for the principal effect.
**Wu, 2025 [48]**	NR	72-h morphine-equivalent consumption	72 h	Approximately 23% lower opioid use; rebound pain 13.0% vs 29.1%; RR 0.46	NR	Rebound pain was secondary; pediatric non-orthopedic population; applicability to adult orthopedic surgery is indirect.
**Lee, 2023 [39]**	71	Change in pain score before vs after ISB resolution	7 days	Pain increase 4.9 vs 4.0; rebound pain 44.4% vs 20.0%; block duration 19.9 vs 15.1 h	NR	Single-centre comparison of routes without a no-dexamethasone group; modest sample size.
**Charier, 2021 [49]**	NR	Postoperative sleep architecture	Postoperative nights 1-3	REM suppression and subsequent REM rebound occurred in GA groups; regional technique preserved sleep architecture	NR	Not a rebound-pain study; indirect outcome and limited relevance to the review question.

**Li, 2024 [23]**	NR	Incidence/severity of rebound pain	36 h	Longer block and lower pain at 12-20 h, but higher rebound-pain incidence and scores; higher pain at 32-36 h	NR	Small, single-centre trial; abstract/reporting inconsistency regarding the adjuvant; no CI reported.
**Lee, 2023 [10]**	66	Pain during block-resolution period	48 h	Continuous block reduced early rebound pain; total opioid use was not reduced versus single-shot block	NR	Three-arm, modest-sized trial; no blinding reported; short follow-up and multiple pain time points.
**Park, 2025 [20]**	NR	Pain at 12 h / rebound-pain incidence	48 h	VAS 2.7 vs 4.1; rebound pain 18.8% vs 65.7%; lower 48-h fentanyl use	NR	Single-centre surgical-injection intervention; participant/provider blinding may be difficult; CI not reported.
**Huan, 2025 [40]**	NR	Incidence of rebound pain	48 h	Lower rebound-pain incidence; lower pain at 12 and 24 h; delayed rescue analgesia and lower 48-h sufentanil use	NR	Single-centre study; sample size and effect estimates/CI not reported in the available abstract.
**Zeng, 2024 [42]**	NR	Incidence of rebound pain	7 days	Esketamine reduced rebound-pain incidence and 24-48-h pain and sufentanil consumption	NR	Non-orthopedic, single-centre thoracic population; procedure-specific pain trajectory limits transferability.
**Korkusuz, 2023 [14]**	60	24-h tramadol consumption and modified rebound-pain score	48 h	Tramadol 45.17 vs 95.00 mg; lower MRPS at rest and movement	NR	Small, single-centre, non-orthopedic study; modified rebound-pain measure limits comparability.
**Akaravinek, 2023 [24]**	NR	Post-discharge moderate-to-severe pain	12 days	Moderate-to-severe pain 70.0% vs 69.2%; no difference in rebound pain, NRS or function	NR	Rebound pain was secondary; complex background multimodal regimen may dilute the isolated intervention effect.
**Kim, 2023 [44]**	NR	Postoperative pain at 4, 8, 12, 24 and 48 h	48 h	Clinically meaningful benefit only at 8 h (1.9 vs 4.7); no reduction in fentanyl use	NR	Rebound pain was not a standardized primary endpoint; modest sample and multiple time-point comparisons.
**Sellbrant, 2021 [50]**	90	Pain and opioid consumption during days 0-3	3 days	Median NRS 1.0 vs 2.7 and opioid-equivalent consumption 30 vs 85 mg for mepivacaine vs ropivacaine	NR	Single-centre, three-arm trial; rebound pain was inferred from pain trajectory rather than prespecified.
**Kim, 2022 [51]**	68	24-h postoperative IV-PCA analgesic consumption	24 h	Higher morphine-equivalent analgesic use with remifentanil; similar mean NRS	NR	Rebound pain was not the primary endpoint; short follow-up and intervention not designed specifically for rebound pain.

**Woo, 2021 [18]**	60	Change in pain score after ISB resolution	48 h; selected outcomes to 30 days	Pain increase 4.5 vs 6.9; rebound pain 37.1% vs 82.9%	NR	Single-centre, modest sample; limited precision and procedure-specific population.
**Lee, 2021 [52]**	NR	Early postoperative pain	48 h	Rebound-pain timing 36 vs 23 h; rebound pain occurred in 6 patients in each group	NR	Rebound pain was secondary; small event count and no reduction in incidence.
**Nobre, 2025 [13]**	NR	Incidence of rebound pain	NR	Control 41.2%, dexamethasone 28.6%, clonidine 23.3%; P=0.22	NR	Likely underpowered for between-group differences; three arms; CI and exact follow-up not reported in the abstract.
**Kasper, 2025 [53]**	NR	Block duration and rebound-pain profile	24 h	Route altered block fade/resolution but did not clearly reduce pain scores or opioid use	NR	Small, single-centre blinded trial; short follow-up; no no-dexamethasone control.
**Keller, 2025 [54]**	36	Total morphine consumption by 20:00 on POD2	POD2	Morphine 39 vs 24 mg; MD 15 mg (ropivacaine vs placebo); P=0.04	NR	Small, single-centre non-orthopedic trial; rebound pain was explanatory/post hoc rather than a prespecified endpoint.
**Jongkongkawutthi, 2025 [22]**	70	Motor recovery time	NR	Rebound pain 8.6% vs 28.6%; P=0.031; no significant difference in motor/sensory recovery	NR	Rebound pain was secondary; modest sample; follow-up and CI not reported in the abstract.
**Aguilera, 2025 [45]**	NR	Block onset/duration and postoperative analgesia	NR	Longer motor, sensory and analgesic duration with 0.5% bupivacaine; no significant rebound-pain difference reported	NR	Rebound pain was secondary and quantitative rebound-pain results were not reported in the abstract.
**Hillesheim, 2021 [55]**	NR	Early postoperative pain/opioid consumption	12 weeks	ISB reduced day-of-surgery pain/opioids but increased opioid use on POD1-2; cumulative opioid use was similar	NR	Rebound pain was inferred from postoperative trajectory; intervention comparison and cost context limit causal interpretation.

Abbreviations: CI, confidence interval; GA, general anesthesia; ISB, interscalene block; MD, mean difference; NR, not reported; POD, postoperative day; PN, perineural; RR, risk ratio. Effect estimates and confidence intervals are presented as reported in the original publications. No estimates were calculated when the required data were unavailable. The limitations column is descriptive and does not represent a formal risk-of-bias assessment.
